# Resistance or compliance? The impact of algorithmic awareness on people's attitudes toward online information browsing

**DOI:** 10.3389/fpsyg.2025.1563592

**Published:** 2025-08-01

**Authors:** Ying Yuan, Yin Shi, Ti Su, HanXuan Zhang

**Affiliations:** ^1^School of Journalism and Communication, Beijing Normal University, Beijing, China; ^2^Faculty of Education, Beijing Normal University, Beijing, China; ^3^Collaborative Innovation Center of Assessment for Basic Education Quality, Beijing Normal University, Beijing, China; ^4^School of Journalism, Fudan University, Shanghai, China

**Keywords:** algorithmic consciousness, perceived power, compliance, resistance, internal control

## Abstract

**Introduction:**

With the pervasive integration of big data algorithms into daily life, individuals increasingly rely on algorithmic filtering to access information of interest, yet simultaneously face the risk of confinement within “information cocoons.” This study examines how algorithmic awareness influences people's attitudes toward online information browsing.

**Methods:**

Using a 2 × 2 mixed design (algorithmic awareness × browsing purpose), this study reveals the mediating roles of perceived power and internal control. Participants performed simulated information-seeking tasks where all content was randomly presented (eliminating actual algorithmic bias). Mediating roles of perceived power and internal control were analyzed using PROCESS macro.

**Results:**

Algorithmic awareness significantly increases people's compliance, especially when they browse online information with a certain purpose, despite all information in the experiment being presented randomly. Algorithmic awareness increases people's sense of power and internal control over information, which, in turn, increases algorithmic compliance behavior.

**Discussion:**

Results reveal that algorithmic awareness reshapes user behavior through psychological empowerment, even in algorithm-neutral environments. By studying the influencing factors of mobile new media users' compliance and resistance to algorithms, this study is helpful in assisting users to eliminate their state of technological unconsciousness and achieve good algorithmic use.

## 1 Introduction

As big data technology continues to evolve, algorithms are increasingly becoming an integral part of various aspects of people's daily lives online (Willson, 2017). Human production, living, and social interactions exhibit new characteristics with the aid of intelligent technology. As a new complimentary approach to decision-making, algorithms reconstruct the underlying operational logic of information production and dissemination and are consequently becoming an important bridge for humans to perceive and understand the diverse meanings of the world (Su, 2023). On the one hand, algorithms, by filtering information and constructing models, reduce the cognitive burden and enhance cognitive efficiency, potentially resulting in advantages in terms of decision-making speed and efficiency (Peng, 2021a). This greatly facilitates personal life and improves the efficiency of daily living. On the other hand, people are aware that if decision-making becomes increasingly entrenched in the patterns created by algorithms, human imagination and creativity may also wither (Peng, 2021b). Research at different levels has shown that people may exhibit resistance to algorithmic recommendations due to concerns such as privacy breaches. However, the factors underlying why individuals exhibit divergent attitudes toward algorithmically recommended information, with some complying and others resisting, remain unclear.

In recent years, scholars from related fields such as communication, psychology, and economics have provided explanations for individuals who make different algorithmic decisions from various perspectives, including algorithmic awareness (Lü et al., 2020), perceived conspiracy (Ghanbarpour et al., 2022), privacy concerns (Bleier and Eisenbeiss, 2015) and notably, algorithmic empowerment (Laukyte, 2022). Algorithmic empowerment refers to the process and state where users perceive enhanced agency, control, or efficacy in their interactions with algorithmic systems (Hodges and Trace, 2023). This perception can stem from understanding how algorithms work (awareness), feeling capable of influencing or navigating algorithmic outcomes, or leveraging algorithmic tools to achieve personal goals. It represents a critical endogenous experience where the user feels less subject to the algorithm's dictates and more like an active participant or even a co-controller. Researchers have found that perceived power can impact an individual's physiological levels, psychological tendencies, and behavioral expressions in multiple aspects (Magee and Galinsky, 2008). Thus, algorithmic awareness and browsing purposes specifically influence people's attitudes and behaviors toward algorithms. However, the precise pathways through which variables like awareness and context (e.g., browsing purpose) foster or hinder this sense of empowerment, and how this empowered state subsequently translates into specific decision-making behaviors (compliance or resistance), remain poorly understood and constitute a significant gap this study addresses.

The theory of internal and external control posits that individuals have different explanations for the source of control over events and their outcomes in their lives (Davis and Phares, 2010). Internals tend to believe that they can control the progression and outcomes of events, whereas externals believe that the results of most things in life are caused by external forces beyond personal control (Gerstorf et al., 2019; Julian and Katz, 1968). Thus, by examining perceived power and locus of control as mediators, we directly probe core components of this algorithmic empowerment experience.

While extant research has effectively mapped the manifestations of algorithmic resistance/compliance (Velkova and Kaun, 2021) and identified key antecedent variables, critical mechanistic and methodological gaps persist. First, there remains a limited understanding of the psychological pathways through which algorithmic awareness translates into divergent behavioral outcomes—compliance vs. resistance. The roles of core endogenous experiences like perceived power (Magee and Galinsky, 2008) and locus of control (Julian and Katz, 1968), which are theoretically central to agency and decision-making under external influence, remain underexplored as potential mediators in this context. Second, current explanations often lack granularity in explaining why individuals under similar algorithmic exposure exhibit fundamentally different behavioral choices. Methodologically, while valuable insights have been generated through qualitative approaches and survey-based studies (Lv et al., 2022; Zhang et al., 2024; Huangfu, 2021), these prevalent research designs may present certain limitations for addressing specific aspects of our research questions.

This study aims to investigate individual algorithmic decision-making behaviors from the perspective of endogenous experiences. By using experimental research methods, with users' awareness of algorithms and the presence or absence of a browsing purpose as independent variables and individual levels of power perception and internal-external control as mediating variables, this study explores the underlying mechanisms that influence algorithmic decisions. This approach can help broaden our understanding of the algorithmic decision-making process and provide insights into how individuals can better achieve the “benevolent use of algorithms.”

## 2 Literature review

### 2.1 Algorithmic awareness and online browsing behavior

The concept of “algorithmic awareness” was proposed by Hargittai et al. (2020) and is defined as an individual's perception and speculation regarding the existence of algorithms, their operational logic, and their social impact during the process of algorithmic practice. Some studies suggest that algorithmic awareness refers to the accuracy of people's perception of algorithmic behaviors in specific media environments and the impact of algorithms on user consumption and experience of media content (Zarouali et al., 2021). This perception encompasses aspects such as the presence of algorithms, how they operate, and the potential risks that they may pose (Hong and Chen, 2022). In this study, algorithmic awareness is conceptualized as an individual's perception of the existence of algorithms in the media environment, which represents a comprehensive media literacy capability.

Because of the powerful opacity of algorithmic technology, most individuals are in a state of “algorithmic unconsciousness” during usage, which makes it difficult for them to keenly perceive the presence of algorithms and the various threats that they pose. Therefore, having algorithmic awareness becomes a necessary prerequisite for dealing with algorithms.

Research has shown that algorithmic awareness affects people's online browsing behavior. In the process of developing algorithmic awareness and evaluating and reflecting on algorithmic mechanisms, users exhibit two types of behavior based on their interaction with algorithms (Tang et al., 2022; Chen and Lü, 2022; Ettlinger, 2018). The first is resistance, where users refuse to cooperate with algorithmic rules and engage in “algorithmic resistance” by deliberately disrupting or disabling algorithmic systems. The second is cooperation, where users actively utilize algorithmic rules to efficiently and conveniently obtain more satisfactory results, exhibiting “algorithmic compliance” (Zhao and Lin, 2023; Hong and Chen, 2022). Karizat et al. (2021) found that TikTok users engaged in behaviors such as deliberately clicking on content that they were not interested in, following more creators with cross-domain identities, and posting videos that aligned with their real-world social identities to resist the marginalization of their social identities by the algorithms. Hong and Chen (2022) approached this issue from the perspective of endogenous experiences and discovered, through the use of in-depth interviews and grounded theory methods, that the factors that influence users' algorithmic compliance behavior include the relevance of information, the nature of information appeals, experience assessment, and boundary perception. When individuals identify with algorithmic information and experience positive perceptions and a sense of self-efficacy, they are more likely to trust and accept the recommended content from algorithms, which leads to algorithmic collaborative behavior (Bao and Zhao, 2023). Algorithm awareness mediates the relationship between social/parasocial motivations and compulsive TikTok use. While amplifying engagement behaviors, it paradoxically fails to mitigate platform fatigue—highlighting its dual role as both an enabler and unintended exacerbator of problematic usage patterns (Wang and Shang, 2024). Concurrently, research reveals a behavioral paradox in algorithmic engagement: users demonstrate heightened awareness of algorithmic manipulation (evidenced by concerns over content oversimplification, commercial exploitation, and political bias), yet exhibit inconsistent resistance behaviors that oscillate between active pushback and passive compliance with curated content (Lin, 2025).

When individuals accept content and products recommended by algorithms, they are, in a sense, making judgments and decisions with the help of algorithms. That is, they are basing their value judgments of content and products on the evaluations of algorithms (Eslami et al., 2015; Hong and Chen, 2022). This process, which is often unconscious, shapes our daily lives (Beer, 2009). Research has shown that the algorithmic ecosystem of traditional search engines heightens users' algorithm awareness, which may lead to algorithm avoidance behaviors, ultimately resulting in a shift in their information-seeking domains (Wan and Xia, 2024). Negative experiential contexts triggering users' algorithm awareness bifurcate into functional—level algorithmic incompetence and mechanism-level algorithmic intrusion. Within these two contextualized algorithmic engagements, users' resistance intentions and resistive behavioral outcomes exhibit incompatibility (Jiang and Xing, 2025).

Therefore, we propose Research Hypothesis 1: Algorithmic awareness significantly affects users' online browsing behavior (H1).

### 2.2 The influence of algorithmic awareness on perceived power

Perceived power is an individual's subjective perception of their ability to influence and control others and to act without reliance on others based on their resources (Anderson et al., 2012; Galinsky et al., 2003). On the one hand, perceived power can reflect an individual's long-term perception of their own power. On the other hand, it is also the psychological feeling of having power or lacking power that an individual experiences in certain specific situations (Jin and Tu, 2018).

The widespread availability of media technology empowers individuals who previously had relatively little power and facilitates their autonomy in accessing, selecting, and disseminating information. “Individuals who have never been ‘seen' in history are now present, and the actions and preferences of individuals who were once blurred are being understood” (Yu and Geng, 2020).

In the process of online empowerment, individuals who have access to and can use the internet can obtain and disseminate information and perspectives through the internet, thereby expanding their capabilities in the real world. Researchers have elaborated on the concept of internet empowerment, suggesting that empowerment can be unfolded in the following four stages: first, at the individual level, which includes the shaping of personal identity and the enhancement of individual skills; second, at the interpersonal level, which encompasses social compensation, the facilitation of cross-cultural communication, and the reduction of stereotypes; third, at the group level, where the internet can aid group members in finding one another, strengthening the collective identity of the group and enhancing the efficiency of group decision-making; and finally, at the level of citizenship, where internet empowerment can increase the accessibility of information and promote civic political participation, oversight, and the capacity to influence government decisions. As research progresses, scholars believe that internet empowerment will lead to new power hierarchies in online society. Ordinary individuals have the opportunity to become influential platform opinion leaders (key opinion leaders, KOLs) within online communities or platforms, endowing them with greater power in the social structure of the virtual space and even “leading to changes in the entire social power structure” (Liang and Liu, 2013).

Algorithmic perception refers to the understanding and conjecture that ordinary users form about the existence, operational logic, and social impact of algorithms during the algorithmic practice process (Hargittai et al., 2020). Analyzing algorithmic perception from the user's perspective and exploring the impact of algorithms on user cognition and behavior are effective ways to reveal the black box of algorithms based on comprehensibility.

Research has shown that through specific environmental cues such as platform usage scenarios and recommended content, users can perceive the existence of algorithms, and algorithmic perception influences users' cognition and feedback behaviors (Yan, 2022). Positive algorithmic perception can effectively enhance user satisfaction with algorithmic services (Shin and Park, 2019). Shin et al. (2020) proposed an algorithmic acceptance model, which verified that user algorithmic perception enhances the perceived usefulness and perceived ease of use of algorithmic services through trust, thereby affecting users' attitudes toward and willingness to use algorithmic services. When users believe that they can understand the basic functions and operating rules of algorithms and can engage in predictable and effective interactions with the system, they will experience a greater sense of information control (Shin and Park, 2019). Scholars have already suggested that enhancing users' sense of information control can reduce their privacy concerns (Morimoto, 2021). Research advances a familiarity-breeds-trust framework, positing that sustained engagement with dating apps cultivates algorithm awareness, which in turn reinforces trust in algorithmic recommendations by empowering users' perceived control over their digital interactions (Hu and Wang, 2024). University students with heightened algorithm awareness—particularly those engaged in algorithm-driven social media information sharing—demonstrate increased frequencies of information dissemination, alongside greater tendencies toward both internalized and externalized maladaptive behaviors (Sun et al., 2024). Research indicates that college-aged users generally adopt a pragmatic stance toward personalized recommendation systems, actively engaging in algorithmic resistance and domestication to restructure content and optimize information sorting—ultimately maximizing the platform's utility for personalized ends (Zhao and Zhou, 2023). Algorithm awareness represents a significant breakthrough beyond the critical algorithm control research paradigm. It emphasizes how ordinary users—devoid of specialized expertise—perceive, comprehend, and recognize algorithms within algorithmic contexts. This form of subjective perception rooted in algorithmic environments demonstrates the potential to shape and influence subsequent behavioral responses (Yan, 2025).

Therefore, we propose Research Hypothesis 2: Algorithmic perception affects the level of perceived power in different contexts (H2).

### 2.3 The influence of perceived power on online browsing behavior

The level of individual perceived power affects cognition and behavior (Park et al., 2015). When external circumstances may pose a threat to an individual's perceived power, individuals with high perceived power are more inclined to cooperate with others and sacrifice personal interests to maintain group interests (Lammers et al., 2008), and they are more attentive to situational factors, which, in turn, influence their prosocial or group-oriented behaviors. The situational focus theory of power suggests that high power perception enables individuals to have greater selectivity and flexibility in information processing to focus on the task at hand and suppress interference from irrelevant information. To the contrary, individuals with low power perception find it difficult to distinguish between relevant and irrelevant information and are unable to concentrate on the task at hand (Galinsky et al., 2008; Guinote, 2007; Weick and Guinote, 2008). Concentration further leads to an underestimation of time intervals, creating a perception that time passes more quickly (Zakay and Block, 1997, 2004). Beer (2017) indicated that the concept of algorithms can play a powerful role in shaping decisions and influencing behavior, thereby constituting social power and discursive status. On this basis, some scholars have proposed that people adopt confrontational strategies to achieve a balance in the human-algorithm relationship and coexistence between humans and machines (Wang, 2023). However, Su (2023) holds that algorithms in the public sphere face the risk of structural imbalance, which specifically includes “subject alienation” weakening public participation in production and communication, the “traffic-first” operational logic intensifying the loss of value rationality, and the solidification and immersion of “information cocoons” dissolving publicness.

According to the locus of control theory of power (Rotter, 1966), the perceived sense of power that individuals have further influences their sense of internal control and external control. Perceived control, as a fundamental need for power, refers to the belief that individuals have in their ability to determine their own internal states and external behaviors, to influence their surrounding environment, and to achieve expected outcomes (Wallston et al., 1987). It is the perception that individuals have about their ability to control events to the extent and degree that they can or cannot control them (Burger, 1989; Frazier et al., 2011). The locus of control theory posits that individuals have different explanations for the source of control over events and outcomes in their lives. For internals, the locus of control lies within the individual; they believe that the outcomes of most events in their lives are determined by the effort that they put into accomplishing these things, and they trust their ability to control the progression and outcomes of events. In contrast, for externals, the locus of control is external to the individual; they believe that the results of most things in their lives are caused by external forces beyond their control, such as societal arrangements, destiny, and luck. They may believe that their personal efforts are futile in the face of these larger forces.

Therefore, we propose Research Hypothesis 3: The level of an individual's perceived power affects their online browsing behavior (H3). In different browsing contexts, internal control (H3a) and external control (H3b) play different mediating roles.

Building upon the conceptual synthesis, this study employs a laboratory experiment to examine the mechanisms underlying individuals' reactions to algorithmic awareness during online information-seeking. Critically, to ensure the ecological validity of the simulated browsing environment, we first conducted a pilot study to curate affectively neutral reading materials sourced from dominant mobile-era new media platforms. These validated materials were then utilized within the main laboratory simulation to reveal the mediating roles of perceived power and internal control. This study directly addresses the unresolved question of why certain individuals exhibit compliance with algorithmic recommendations while others demonstrate resistance.

## 3 Pilot study

### 3.1 Experimental purpose

To avoid the influence of emotions and social expectations on the cognitive processing of algorithmic push materials by new media users and to ensure that participants do not experience significant emotional fluctuations because of the content of the materials, a pilot study was conducted to select a certain number of neutral reading materials as the push materials in the formal experiment. Familiarity with the content and the emotional valence that it evoked were also assessed.

### 3.2 Experimental process

First, through an open-ended questionnaire, the expert panel of the research group collected reading materials that university students frequently browsed on their daily social media platforms. The expert panel selected two categories from commonly used new media platforms in the mobile era, such as Xiaohongshu (Rednote), which focuses on daily life, film and television works. A total of 158 reading materials were collected, and each included ~100 words of reading text and one corresponding image.

A total of 199 participants were then randomly recruited from a university to evaluate the familiarity and emotional valence of the collected reading materials. The instructions were as follows: “Hello, fellow student. We are doing xx research and invite you to participate in our study… Thank you for participating in this experiment. Below are some diverse reading materials. Please rate the degree of your liking the information and the judgment of the content based on your preferences. Everyone has different personalities, so there are no right or wrong answers. Please answer based on your actual situation.” The participants were then asked to rate their liking of the information on a scale from “1 = Dislike very much” to “7 = Like very much.” The positivity/negativity of the content conveyed by the information was rated on a scale from “1 = Very negative” to “7 = Very positive,” and their familiarity with the content described in the information was rated on a scale from “1 = Very unfamiliar” to “7 = Very familiar.”

### 3.3 Results

Among the 199 participants who evaluated the neutral reading materials, there were 111 females and 88 males, with an average age of 31.71 years (*SD* = 9.35). The mean values for the familiarity and emotional valence of the 158 reading materials were calculated (*M*_*Familiarity*_ = 6.03, *SD*_*Familiarity*_ = 0.58; *M*_*Liking*_ = 5.85, *SD*_*Liking*_ = 0.35; *M*_*Positivity*/*Negativity*_ = 5.78, *SD*_*Positivity*/*Negativity*_ = 0.36). Thirty sets of text and corresponding images were selected as the most suitable neutral reading materials, with 15 sets related to daily life and 15 sets related to film and television sharing. These materials were liked by the participants to an average degree, conveyed content of a positive nature to an average degree, and had a relatively small difference in content familiarity.

There was no significant difference in familiarity with the reading materials between the daily life group (*M* = 6.17, *SD* = 0.54) and the film and television sharing group (*M* = 5.91, *SD* = 0.57), *t*_(28)_ = 1.28, *p* = 0.21, Cohen's *d* = 0.47, effect size (*r*) = 0.23. In terms of the emotional valence caused by the reading materials, there was no significant difference between the degree to which the daily life group materials were liked by the participants (*M* = 5.55, *SD* = 0.17) and the film and television sharing group (*M* = 5.60, *SD* = 0.22), *t*_(28)_ = −1.09, *p* = 0.28, Cohen's *d* = −0.40, effect size (*r*) = −0.20. There was also no significant difference in the positivity of the content conveyed by the images in the daily life group (*M* = 5.48, *SD* = 0.21) and the film and television sharing group (*M* = 5.58, *SD* = 0.29), *t*_(28)_ = −0.67, *p* = 0.51, Cohen's *d* = −0.25, effect size (*r*) = −0.12.

Therefore, we determined the neutral reading materials for the formal experiment's purposeless group (daily life) and purposeful group (film and television sharing) and that the reading materials themselves would not cause significant emotional fluctuations in the participants.

## 4 Formal experiment

### 4.1 Experimental purpose and participants

The purpose of this study is to examine why people engage in resistance to algorithmic power during new media browsing. The study treats as independent variables whether individuals are aware of algorithms and whether they have a clear browsing purpose.

A 2 (algorithmic awareness: presence, absence) × 2 (browsing purpose: presence, absence) mixed factorial experimental design is used to investigate the differences in resistance to algorithmic power under different levels of algorithmic awareness and browsing purposes. The presence or absence of algorithmic awareness is a between-subjects variable, while the presence or absence of a clear browsing purpose is a within-subjects variable.

We calculated the required sample size for the study using G^*^power 3.1 (Faul et al., 2007). Following Cohen's guidelines, with a two-way mixed analysis of variance (ANOVA) as the statistical method, an effect size of *f* = 0.2, α = 0.05, 1-β > 0.8, and respective sample sizes of 2 for within-subjects and between-subjects factors, the minimum sample size was 52 participants.

Therefore, we randomly recruited 75 undergraduate students through campus announcements from a university (all of whom had not participated in the pilot study) to participate in the experiment. All participants received 30 yuan upon completion of the experiment.

Participants were randomly assigned to one of two groups (the algorithm awareness group or the no algorithm awareness group) and were informed of the complete procedure accordingly. They could withdraw from the experiment at any time if they felt any discomfort. The purpose of the algorithmic awareness manipulation was explained, and psychological support was provided after they completed the experiment.

As a manipulation check, we included a single-choice question in the post-experiment questionnaire asking participants to select how the information they viewed during the experiment was filtered: “Algorithmically filtered” or “Randomly filtered.” This was used to verify whether the participants correctly understood the experimental instructions. We also used a seven-point Likert scale “To what extent did you believe the information was algorithmically filtered?” as a measure of participants' level of algorithmic awareness.

The data of 7 participants were excluded because they provided incorrect responses (i.e., participants in the algorithm awareness group who answered “randomly filtered” and those in the no algorithm awareness group who answered “algorithmically filtered”), resulting in a final sample of 68 participants, with 10 male participants (15.6%) and 58 female participants (84.4%).

### 4.2 Experimental procedure and materials

#### 4.2.1 Experimental procedure

**Figure 1:**
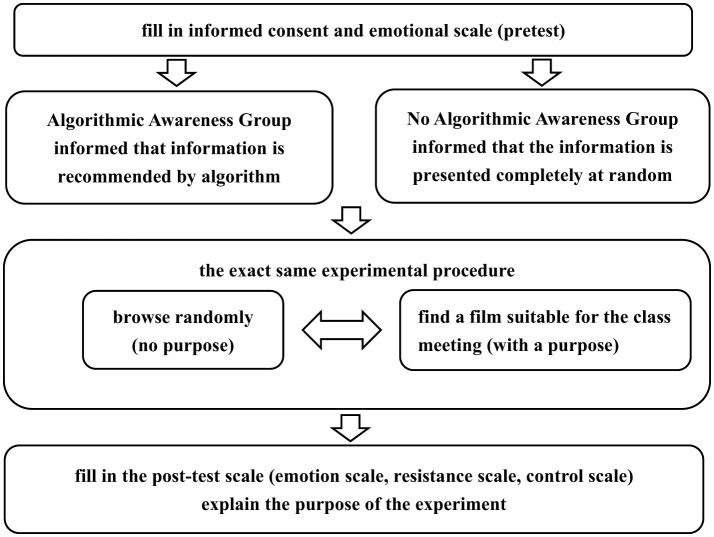
Formal experimental operational flow chart.

The experimental procedure is shown in Figure 1. The participants were first guided to sign an informed consent form upon entering the laboratory. Since users may also be influenced by their own emotions during the behavior process (Geber et al., 2021), that is, there are irrational triggers for users' algorithmic compliance or resistance behaviors, the emotional levels of the participants were measured both before and after the experiment. After the participants had calmed their emotions, they completed an emotional scale based on their current emotional state.

After completing the form, the participants were randomly assigned to the “algorithmic awareness” group or the “no algorithmic awareness” group. They were then led into the eye-tracking laboratory by the experimenter, where they began the information browsing task using an identical experimental procedure. However, the “algorithmic awareness” group was informed by the experimenter that “the information is pushed based on your behavioral habits according to the algorithm,” to activate algorithmic awareness, whereas the “no algorithmic awareness” group was told by the experimenter that “the information is presented completely randomly.”

Both groups of participants were required to complete “purposeful” and “purposeless” information browsing tasks, with the order of presentation and the number of tasks being random for both of these tasks. Specifically, during the “purposeless” task, the experimenter asked the subject to “wait a few minutes first, the responsible teacher will be here soon. Feel free to browse the information on the screen, press the J key to go to the next page.” This was followed by another experimenter entering the laboratory and instructing the subject to “find a movie suitable for screening at a class meeting, press the J key to go to the next page” to complete the “purposeful” task. The other half of the participants were first informed to find a movie suitable for a class meeting; then, they waited for a few minutes and browsed information randomly.

After the experiment, the experimenter asked the participants to complete the post-experiment emotional rating scale, algorithmic compliance scale, and sense of control scale, among others. Finally, the experimenter explained the purpose of the experiment to the participants and carefully inquired about their experiences to ensure that the experiment had not caused any harm to the participants.

The experimental procedure was developed using E-Prime 3.0 and consisted of 15 trials with a browsing purpose and 15 trials without a browsing purpose. The materials in each trial were the neutral reading materials selected from the pilot study. After the subjects completed browsing the material, they pressed the J key to proceed to the next page. Both the trials with a browsing purpose and those without a browsing purpose were randomly presented in blocks, with each having an equal probability of 50% in terms of their sequence of appearance.

#### 4.2.2 Experimental materials

##### 4.2.2.1 Emotional scale

Based on the revised emotional assessment scale by Li et al. (2013), a secondary revision was conducted. The scale includes seven emotional words: excitement, calmness, happiness, tension, relaxation, anxiety, and sadness. The scoring method uses a seven-point Likert scale, with 1 indicating “none” and 7 indicating “very much.” The scale has good reliability indicators (Cronbach's α = 0.85) and satisfactory validity.

##### 4.2.2.2 Level of compliance

The compliance level of the participants during the “purposeful browsing” and “purposeless browsing” stages was measured using a self-developed scale. The scale consists of 5 items scored with a seven-point Likert scale, with 1 indicating “Strongly Disagree” and 7 indicating “Strongly Agree.” The specific items include (1) “I like the browsing materials presented;” (2) “If time permits, I would continue browsing;” (3) “I want to see more materials;” (4) “I like this information push method;” and (5) “I like this part of the experimental procedure.”

##### 4.2.2.3 Level of perceived power

The subjective sense of power of individuals was measured using the revised General Perceived Power Scale by Anderson and Galinsky (2006). The scale was scored with a seven-point Likert scale, with 1 indicating “Strongly Disagree” and 7 indicating “Strongly Agree.” The specific items include statements such as “I feel I have a great deal of power,” “I can make others do what I want,” “I can get others to listen to me,” and “As long as I want to, I can make decisions on my own.”

##### 4.2.2.4 Levels of internal and external control

Based on Levenson's (1981) Perceived Control, Chance, and Luck Scale, which categorizes psychological control into three components—internal control, other control, and chance—and corresponds to three subscales, the study adjusted some of the wording to fit the experimental context and used these subscales to assess the levels of internal and external control. The internal control subscale was used to measure the sense of internal control, while the other control subscale was used to measure the sense of external control. Both were evaluated with a seven-point Likert scale, with 1 indicating “Strongly Disagree” and 7 indicating “Strongly Agree.”

## 5 Results

### 5.1 Level of emotion

First, a paired samples *t*-test was conducted to statistically analyze the differences in emotional levels before and after testing the 68 participants. The results, as shown in Table 1, indicate that there were no significant differences in positive emotions (happiness and excitement), neutral emotions (calmness and relaxation), or negative emotions (anxiety, sadness, and tension) between the pre-test and post-test, *ps* > 0.05.

**Table 1:** Differences in emotion level measured before and after the intervention.

**Emotion**	**Pre-test (*SD*)**	**Post-test (*SD*)**	**Significance level**
Excitement	2.49 (±1.45)	2.47 (±1.44)	*t*_(67)_ = 0.06, *p* = 0.95, Cohen's *d* = 0.01
Pleasure	4.65 (±1.24)	4.91 (±1.24)	*t*_(67)_ = −1.25, *p* = 0.22, Cohen's *d* = −0.15
Peace	5.12 (±1.40)	5.13 (±1.38)	*t*_(67)_ = −0.07, *p* = 0.95, Cohen's *d* = −0.01
Relaxation	5.02 (±1.40)	5.43 (±1.32)	*t*_(67)_ = −1.65, *p* = 0.10, Cohen's *d* = −0.20
Tension	2.18 (±1.33)	1.78 (±1.21)	*t*_(67)_ = 1.90, *p* = 0.06, Cohen's *d* = 0.23
Anxiety	2.13 (±1.33)	1.93 (±1.35)	*t*_(67)_ = 0.90, *p* = 0.37, Cohen's *d* = 0.11
Sadness	1.37 (±0.84)	1.28 (±0.81)	*t*_(67)_ = 0.64, *p* = 0.52, Cohen's *d* = 0.08

### 5.2 Level of algorithmic awareness

We conducted an independent sample *t*-test to check whether the level of algorithmic awareness was different between the two groups. Participants in the algorithmic awareness group reported they are more believe the information was algorithmically filtered in the algorithmic awareness group (*M* = 4.69, *SD* = 1.85) than those in the no algorithmic awareness group (*M* = 2.50, *SD* = 1.08), *t*_(66)_ = 5.882, *p* < 0.001, Cohen's *d* > 1, which suggested that the algorithmic awareness had been successfully activated.

### 5.3 Level of compliance

A two-way mixed ANOVA was conducted with the level of compliance, with information as the dependent variable and “algorithmic awareness” and “browsing purpose” as the independent variables.

**Figure 2:**
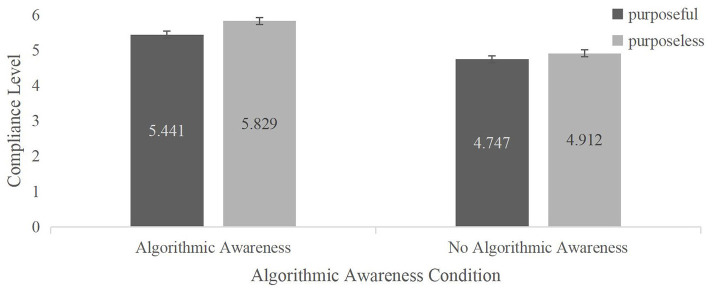
The effect of algorithmic awareness on compliance level scored with or without a browsing purpose. Error bars represent ±1 SEM.

The results, as shown in Figure 2, indicate a significant main effect for browsing purpose, *F*_(1, 66)_ = 7.83, *p* = 0.01, η^2^ = 0.11. When people browse information without a specific purpose, their level of compliance with the information (*M* = 5.09, *SD* = 0.13) is significantly lower than when they have a specific purpose (*M* = 5.37, *SD* = 0.14). There is also a significant main effect for algorithmic awareness, *F*_(1, 66)_ = 10.61, *p* < 0.01, η^2^ = 0.14. When people believe that the information is recommended by algorithms, their level of compliance with the information (*M* = 5.36, *SD* = 0.18) is significantly higher than when they believe that the information is randomly pushed (*M* = 4.83, *SD* = 0.18). The interaction effect between browsing purpose and algorithmic awareness is not significant, *F*_(1, 66)_ = 1.28, *p* = 0.26, η^2^ = 0.02.

A simple effects analysis was conducted on the interaction between having or not having a browsing purpose and being aware or not being aware of being manipulated. When people believe that the information is pushed by algorithms, their level of compliance with the information obtained during purposeful browsing (*M* = 5.83, *SD* = 0.19) is significantly higher than their level of compliance with the information obtained during purposeless browsing (*M* = 5.44, *SD* = 0.19), *F*_(1, 66)_ = 7.72, *p* = 0.01, η^2^ = 0.11. When people believe that information is randomly pushed, the difference in compliance levels between purposeful browsing (*M* = 4.75, *SD* = 0.19) and purposeless browsing (*M* = 4.91, *SD* = 0.19) is not significant, *F*_(1, 66)_ = 1.39, *p* = 0.24, η^2^ = 0.02.

### 5.4 The mediating effects of perceived power and sense of control

#### 5.4.1 Compliance with information during purposeful browsing

By using the regression-based mediation process analysis method proposed by Preacher and Hayes, with Hayes' bootstrap test procedure developed in the SPSS Process plugin, and setting the sample size (bootstrap samples) to 5,000, the theoretical Model 4 pre-set in the plugin was tested.

**Figure 3:**
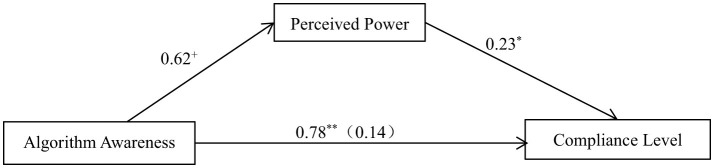
The mediating effect of sense of power on algorithmic consciousness and compliance level (purposeful). ^**^p < 0.01, ^*^p < 0.05, ^+^*p* < 0.1.

With algorithmic awareness as the independent variable, the level of compliance with information during purposeful browsing as the dependent variable, and perceived power as the mediating variable, the results show that the following (Figure 3). (1) Algorithmic awareness has a significant positive effect on the level of compliance with information during purposeful browsing, β = 0.78, *SE* = 0.27, *p* = 0.01. Consistent with the results of the two-way ANOVA, compared with when people believe that the information is randomly presented, the compliance level is higher when people are aware that the information is pushed by algorithms. (2) Algorithmic awareness has a marginally significant positive effect on the level of perceived power, β = 0.62, *SE* = 0.32, *p* = 0.06. Compared with when people believe that the information is randomly presented, the level of perceived power is higher when people are aware that the information is pushed by algorithms. (3) The level of perceived power has a significant positive effect on the level of compliance with information during purposeful browsing, β = 0.23, *SE* = 0.10, *p* = 0.03. When the level of perceived power is higher, the compliance level is higher with information obtained during purposeful browsing.

Using the bias-corrected non-parametric percentile bootstrap method, the mediating effect of perceived power on the influence of algorithmic awareness on compliance level was tested, with a confidence interval set at 95%. The mediating effect of “algorithmic awareness → perceived power → compliance level” was significant (*Effect* = 0.14, *SE* = 0.10, 95% CI = [0.00, 0.42]), which indicates that perceived power partially mediated the influence of algorithmic awareness on the level of compliance with information during purposeful browsing and the mediating effect of perceived power accounted for 18% of the total effect.

**Figure 4:**
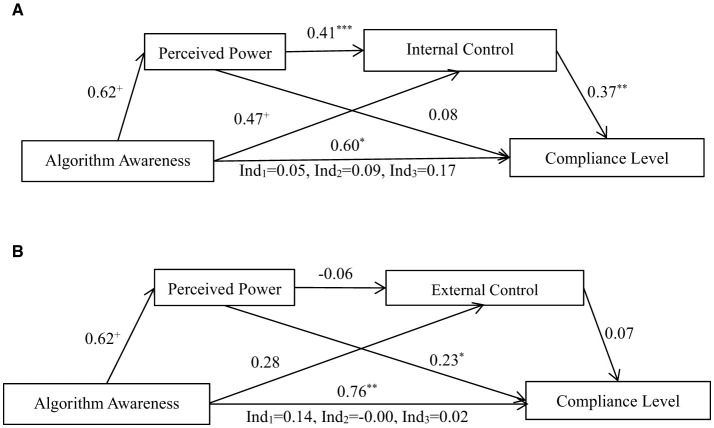
**(A)** An analysis of the chain mediating effects of sense of power and internal control. **(B)** An analysis of the chain mediating effects of sense of power and external control. ***p < 0.001; **p < 0.01; *p < 0.05; ^+^*p* < 0.1.

According to the locus of control theory of power (Rotter, 1966), the perceived sense of power that individuals have further influences their sense of internal control and external control. Therefore, the bootstrap test procedure developed by Hayes was used to test the pre-set theoretical Model 6 in the SPSS Process plugin. The sampling sample size (bootstrap samples) was set at 5,000, and chain mediating effects were tested for “algorithmic awareness → perceived power → internal control → compliance level” and “algorithmic awareness → perceived power → external control → compliance level,” with the results shown in Figures 4a, b.

The results indicate that the chain mediating effect of “algorithmic awareness → perceived power → internal control → compliance level” is established, with *Effect* = 0.09, *SE* = 0.07, 95% CI = [0.01, 0.29], whereas the chain mediating effect of “algorithmic awareness → perceived power → external control → compliance level” is not established, with *Effect* = −0.00, *SE* = 0.01, 95% CI = [−0.06, 0.01]. Therefore, when people believe that information is pushed by algorithms, their level of perceived power increases, which, in turn, enhances their sense of internal control rather than external control, thereby leading to compliance with algorithmic information.

Ind_1_ represents the mediating effect of “algorithmic awareness → perceived power → compliance level,” Ind_2_ indicates the chain mediating effect of “algorithmic awareness → perceived power → internal (external) control → compliance level,” and Ind_3_ denotes the mediating effect of “algorithmic awareness → internal (external) control → compliance level.”

#### 5.4.2 Compliance with information during purposeless browsing

**Figure 5:**
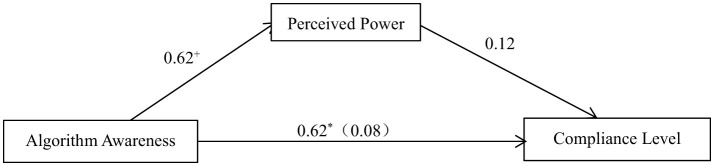
The mediating effect of perceived power on the influence of algorithmic awareness on compliance level (purposeless). *p < 0.05; ^+^*p* < 0.1.

Using the regression-based mediation process analysis method (Hayes, 2013), the bootstrap test procedure developed was employed to test the pre-set theoretical Model 4 in the SPSS Process plugin, with the sample size (bootstrap samples) set at 5,000 for testing (Figure 5).

With algorithmic awareness as the independent variable, the level of compliance with information during purposeless browsing as the dependent variable and perceived power as the mediating variable, the results show that the following. (1) Algorithmic awareness has a significant positive effect on the level of compliance with information during purposeless browsing, β = 0.62, *SE* = 0.27, *p* = 0.02. Consistent with the results of the two-way ANOVA, compared with when people believe that the information is randomly presented, the compliance level is higher when people are aware that the information is pushed by algorithms. (2) The level of perceived power does not have a predictive effect on the level of compliance with information during purposeless browsing, β = 0.12, *SE* = 0.10, *p* = 0.22.

Using the bias-corrected non-parametric percentile bootstrap method, the mediating effect of perceived power on the influence of algorithmic awareness on compliance level was tested, with a confidence interval set at 95%. The mediating effect of “algorithmic awareness → perceived power → compliance level” was not significant (*Effect* = 0.08, *SE* = 0.09, 95% CI = [−0.04, 0.33]). This result indicates that the mediating role of perceived power in the influence of algorithmic awareness on the level of compliance with information during purposeful browsing is not established.

## 6 Discussion and conclusion

This study revealed that when individuals browse online information recommended by algorithms, whether or not there is a purpose, algorithmic awareness significantly affects an individual's compliance level with algorithms. That is, when an individual's algorithmic awareness is stronger, an individual is more willing to use algorithmic recommendation mechanisms to assist in information decision-making activities. In particular, when individuals purposefully browse information, they experience a sense of power over their browsing behavior at a psychological level.

### 6.1 Algorithmic awareness and the shifting boundaries of resistance and compliance

Based on the fundamental role of algorithmic awareness, the sense of power within individuals triggers a strong sense of internal control, which leads them to believe that they have the ability to control algorithms and use them as tools to assist in decision-making. Therefore, individuals exhibit high levels of algorithmic compliance and are willing to continuously browse information recommended by algorithms. In this experiment, there was no significant difference in the emotional measurements of individuals before and after reading the experimental materials, indicating that the experimental materials did not produce a noticeable positive or negative stimulus on the participants' emotions. Positive emotions such as happiness, calmness, relaxation, and neutral emotions remained at high levels. Whether individuals browse algorithmically recommended information with a purpose or not, their perception of algorithms facilitates their algorithmic compliance behavior, and high levels of positive or neutral emotions are not negatively affected by algorithms and their recommended content.

This study found that when individuals face task-driven purposeful browsing and perceive the existence of algorithmic recommendations, under the influence of their sense of power and the resulting sense of internal control, individuals do not experience strong anxiety or resistance toward algorithms but, on the contrary, exhibit high compliance with the information recommended by algorithms. The recommendation algorithms themselves and the information that they provide do not cause significant emotional fluctuations in individuals. Instead, algorithms are seen as rational tools, and the information recommended by algorithms is considered the result of self-screening. Based on the previously high levels of positive and neutral emotions, individuals exhibit continuous browsing behavior, which reflects a compliant attitude toward algorithms.

Under the condition of purposeful browsing, individuals' perceived power plays a role, and their high sense of internal control over algorithms reflects emotions such as happiness and relaxation. At the practical level, individuals delegate their decision-making power to algorithms, which promotes the occurrence of compliant behavior. The content filtering function and recommendation mechanism of algorithms, with their high efficiency and convenient operation, meet people's needs for purposeful browsing, stimulate the motivation for the continuous reading of algorithm-recommended information, and lead to a high level of compliant psychology in people. By strategically embedding explanatory cues during purposeful browsing, such as displaying “Recommended based on your history” when users revisit curated content, platforms may transform passive algorithmic experiences into conscious reflection moments. Under the condition of purposeless browsing, the mediating effect is not significant. Users may rely more on external cues (e.g., information order), weakening internal control mechanisms (Zarouali et al., 2021). This passive mode of engagement reflects a reduced threshold for heuristic processing: algorithmic recommendations function less as decision aids and more as cognitive substitutes, diminishing users' critical evaluation of content. Consequently, behavioral compliance stems not from delegated agency, but from cognitive offloading and attentional inertia, wherein algorithmic pathways default into low-effort browsing trajectories.

This study also revealed that when individuals engage in random information browsing activities, their awareness of algorithms still triggers a sense of power. However, at this time, perceived power does not have a significant effect on individuals' level of compliance with algorithms. This is because people's perception of external control is at play, with algorithms becoming intruders that affect people's information decisions, which leads to psychological alertness and a shift from compliance to caution. This study synthesizes algorithmic awareness, perceived power, and locus of control, highlighting their interplay in shaping users' power negotiation within algorithmic contexts. Collectively, their integration refines theoretical frameworks by capturing the reciprocal dynamics between individual agency and systemic power structures.

### 6.2 How perception of power acts on algorithmic awareness

As users of the internet, whether they are reading social media, searching for information via search engines, or browsing shopping websites, people are constantly influenced by recommendation algorithms and exist in a virtual environment constructed of algorithmically recommended information. This information shapes people's cognition and attitudes and deeply intervenes in their daily lives. With the development of the new media era, the power of the “gatekeeper” has undergone a transformation, shifting from traditional editors and gradually becoming mediated by algorithms that determine the types of content and information that people will encounter on the internet. In the long-term practice of media use and the enhancement of media literacy, people have gradually developed direct or indirect awareness of the existence and mechanisms of recommendation algorithms. Algorithms predict individuals' interest preferences based on their past browsing content and behavior or filter information collaboratively according to the common behavioral characteristics of a group of people, enabling people to have personalized experiences in information consumption. When people browse internet information, their awareness of the existence of algorithms leads to judgments about perceived power: is the power to screen information controlled by self-determination or by algorithms? At the same time, people evaluate the value and utility of algorithms and consider whether algorithms can serve as a tool to assist in achieving goals efficiently when people have browsing purposes, thus influencing their compliance with or resistance to algorithms.

Although recommendation algorithms can provide certain conveniences for people, they have predictable negative impacts that can subtly change people's cognitive and behavioral habits. Algorithms create a unique, bizarre bubble world for every user on the internet by filtering out content that users prefer from the vast amount of overloaded information while immersing people and making them enjoy it. The information that individuals encounter within their bubbles is meticulously customized by recommendation algorithms, and everything that users see and hear aligns with their interests. However, individuals find it difficult to gain a true and comprehensive understanding of the real overall environment and appearance of the world and society, and they gradually develop a cognitive pattern centered on themselves. A more extreme result is that people become obsessed with homogenized information and emotions and become rigid and fanatical. They are repeatedly stimulated by homogenized content within an echo chamber, which leads to the polarization of thought and the radicalization of behavior. Therefore, people should treat recommendation algorithms with caution. Faced with the convenience of algorithms, when people delegate the control of information filtering to algorithms, they should strike a balance and maintain a proper scale between human subjective initiative and the instrumental nature of algorithms. Moderate compliance with algorithms does not mean that one's judgment and decision-making ability are completely abandoned. People need to be vigilant against the inertia of thought and avoid excessive reliance on algorithms or even sinking into “algorithmic unconsciousness.” To resist or comply with the utopian world constructed by algorithms, people should firmly uphold the principles of human subjectivity and consciousness. They should strike a balance between the instrumental and value rationalities of recommendation algorithms by enabling algorithms to better meet people's needs for efficient and accurate information browsing while also having a positive and proactive impact on society, culture, and people's values.

### 6.3 Reflections on the barriers between instrumental rationality and value rationality in algorithmic recommendation

As ethical issues associated with artificial intelligence and big data have become prominent, algorithmic power has emerged as a hot topic of academic research (Bucher and Mølster, 2013; Ferrari and Graham, 2021; Van and Scully, 2024). As intermediaries, algorithms not only influence people's cognition and behavior but also construct, regulate, and control various relationships to gradually become a form of power. From an ontological perspective, the existence and dominion of algorithms seem to be invisible and impenetrable, triggering what some refer to as a “control crisis” (Bodó et al., 2018). Technological trust mediators function as black boxes, prioritizing platform objectives over user agency. This dynamic leads users to confuse algorithmic filtering with autonomous choice, eroding genuine decision-making autonomy under the pretense of control. Du and Zhou (2022) began their discussion on user autonomy from the perspective of privacy issues in algorithmic recommendations. In an era of coexistence with algorithms, “information empowerment” aims to form a user-led information flow mechanism by ensuring the subjective rights and interests of individuals in their information acquisition, processing, and usage. This is intended to “compel” algorithmic mechanisms and information processing to be transparent and open through the self-control and self-determination of personal information. In the digital age, the impact of algorithmic recommendation mechanisms on human autonomy is manifested primarily as a “loss of power” crisis over personal information. However, the current “information empowerment” mechanisms have not yet been effectively established and implemented, which leads to personal information freedom being manipulated by algorithmic power (Du and Zhou, 2022). Platform algorithms establish connections between people and content, essentially configuring visibility. Compared with local visibility and media visibility, the configuration of algorithmic visibility is more covert, random, and uncertain (Yi, 2022). Algorithmic recommendations enhance users' instrumental rationality by efficiently matching their preferences with relevant content, thereby facilitating goal-oriented decision-making. However, they may undermine value rationality by narrowing informational exposure and shaping choices based on opaque criteria, potentially diluting critical engagement with broader societal values.

### 6.4 Limitation and future research

While this study employed a controlled experimental design to rigorously test the causal relationships between algorithmic awareness, perceived power, sense of control, and compliance behavior, the limitation of this study must also be considered. Although the results of the independent samples *t*-test showed that the effect of gender on perceived power, internal control, external control, purposeless compliance and purposeful compliance behavior was not significant (see Table 2), participants were predominantly female undergraduate students. This is partly limited by the higher female-to-male ratio in the sampled school, and partly given that the user base of the modeled social media platform, Rednote, is mostly female (Zhao and Zhou, 2023; Chen and Jiang, 2025; Lu and University of Georgia. Music - MA, 2022; Han, 2025). Thus, it necessitates caution in generalizing findings to broader populations with diverse gender representation, ages, occupations, cultural backgrounds, and baseline levels of algorithmic literacy. Future research should actively incorporate these diverse groups to establish the wider applicability and potential boundary conditions of the observed psychological mechanisms.

**Table 2:** The effect of gender on several key outcome variables.

**Variables**	**Male**	**Female**	**Significance level**
Perceived Power	3.53 (±1.41)	3.31 (±1.36)	*t*_(66)_ = 0.45, *p* = 0.65, Cohen's *d* = 0.11
Internal control	4.57 (±1.57)	3.95 (±1.15)	*t*_(66)_ = 1.49, *p* = 0.14, Cohen's *d* = 0.37
External control	3.98 (±0.89)	4.05 (±0.75)	*t*_(66)_ = −0.29, *p* = 0.77, Cohen's *d* = −0.07
Purposeless compliance	4.96 (±0.99)	5.12 (±1.16)	*t*_(66)_ = −0.41, *p* = 0.69, Cohen's *d* = −0.10
Purposeful compliance	5.20 (±1.15)	5.40 (±1.22)	*t*_(66)_ = −0.48, *p* = 0.63, Cohen's *d* = −0.12

The use of carefully curated, affectively neutral reading materials was a deliberate strength to minimize confounding emotional responses and isolate the target variables. However, neutral materials may underestimate users' emotional responses to personalized content. These materials differ from the personalized, often affectively charged content typical of real-world algorithmic feeds. Furthermore, although laboratory controls ensure internal validity, the artificial lab setting suggests opportunities for improving ecological validity. Future studies could replicate experiments with high-involvement content, including incorporating diverse materials, such as simulated short-video platform or news app recommendation interfaces, and content with varying levels of personal relevance, emotional valence, and stakes. This will test the robustness of the effects in environments closer to actual user experience.

## Data Availability

The raw data supporting the conclusions of this article will be made available by the authors, without undue reservation.

## References

[B1] Anderson C. Galinsky A. D. (2006). Power, optimism, and risk-taking. Eur. J. Soc. Psychol. 36, 511–536. 10.1002/ejsp.324

[B2] Anderson C. John O. P. Keltner D. (2012). The personal sense of power. J. Pers. 80, 313–344. 10.1111/j.1467-6494.2011.00734.x 21446947

[B3] Bao L. L. Zhao Y. R. (2023). Research on the factors affecting the manipulation of short video users by algorithms - from the perspective of information technology identification. Modern Publ. 5, 40–54. 10.3969/j.issn.2095-0330.2023.05.005

[B4] Beer D. (2009). Power through the algorithm? Participatory web cultures and the technological unconscious. New Media Soc. 11, 985–1002. 10.1177/1461444809336551

[B5] Beer D. (2017). The social power of algorithms. Inf. Commun. Soc. 20, 1–13. 10.1080/1369118X.2016.1216147

[B6] Bleier A. Eisenbeiss M. (2015). Personalized online advertising effectiveness: the interplay of what, when, and where. Market. Sci. 34, 669–688. 10.1287/mksc.2015.093019642375

[B7] Bodó B. Helberger N. Irion K. Borgesius K. Z. Mller J. Velde B. V. D.. (2018). Tackling the algorithmic control crisis – the technical, legal, and ethical challenges of research into algorithmic agents. Yale J. Law Technol. 19, 133–180.

[B8] Bucher T. Mølster R. (2013). Want to be on the top? Algorithmic power and the threat of invisibility on Facebook. Nordicom Rev. 34, 153–153. 10.1177/1461444812440159

[B9] Burger J. M. (1989). Negative reactions to increases in perceived personal control. J. Pers. Soc. Psychol. 56, 246–256. 10.1037/0022-3514.56.2.246

[B10] Chen S. Y. Jiang X. X. (2025). Performing balanced aspirations through identity capital: a case study of Chinese Ivy League influencers on RedNote. J. Consumer Cult. 25, 341–360. 10.1177/14695405251330768

[B11] Chen Y. Lü X. (2022). The dialectics of control: resistance to short video platform recommendation algorithms by rural youths - an investigation based on the dual mediation path of “rational - irrational”. Shanghai. J. Rev. 7, 71–87. 10.16057/j.cnki.31-1171/g2.2022.07.004

[B12] Davis W. L. Phares E. J. (2010). Internal-external control as a determinant of information-seeking in a social influence situation. J. Pers. 35, 547–561. 10.1111/j.1467-6494.1967.tb01447.x 6079878

[B13] Du Y. X. Zhou M. J. (2022). Building autonomy in a transparent existence: privacy issues and regulatory paths in algorithm recommendations. Future Commun. 6, 10–19. 10.13628/j.cnki.zjcmxb.2022.06.010

[B14] Eslami M. Rickman A. Vaccaro K. Aleyasen A. Vuong A. Karahalios K.. (2015). “I always assumed that I wasn't really that close to [her]” in Proceedings of the 33rd Annual ACM Conference on Human Factors in Computing Systems (New York, NY: ACM), 235–162. 10.1145/2702123.2702556

[B15] Ettlinger N. (2018). Algorithmic affordances for productive resistance. Big Data Soc. 5, 1–13. 10.1177/2053951718771399

[B16] Faul F. Erdfelder E. Lang A. G. Buchner A. (2007). G^*^ Power 3: a flexible statistical power analysis program for the social, behavioral, and biomedical sciences. Behav. Res. Methods 39, 175–191. 10.3758/BF03193146 17695343

[B17] Ferrari F. Graham M. (2021). Fissures in algorithmic power: platforms, code, and contestation. Cult. Stud. 35, 814–832. 10.1080/09502386.2021.1895250

[B18] Frazier P. Keenan N. Anders S. Perera S. Shallcross S. Hintz S. (2011). Perceived past, present, and future control and adjustment to stressful life events. J. Pers. Soc. Psychol. 100, 749–765. 10.1037/a0022405 21299308

[B19] Galinsky A. D. Gruenfeld D. H. Magee J. C. (2003). From power to action. J. Pers. Soc. Psychol. 85, 453–466. 10.1037/0022-3514.85.3.453 14498782

[B20] Galinsky A. D. Magee J. C. Gruenfeld D. H Whitson J. A. Liljenquist K. A. (2008). Power reduces the press of the situation: Implications for creativity, conformity, and dissonance. J. Pers. Soc. Psychol. 95, 1450–1466. 10.1037/a0012633 19025295

[B21] Geber S. Frey T. Friemel T. N. (2021). Social media use in the context of drinking onset: the mutual influences of social media effects and selectivity. J. Health Commun. 26, 566–575. 10.1080/10810730.2021.1980636 34559039

[B22] Gerstorf D. Drewelies J. Duezel S. Smith J. Wahl H. W. Schilling O. K.. (2019). Cohort differences in adult-life trajectories of internal and external control beliefs: a tale of more and better maintained internal control and fewer external constraints. Psychol. Aging 34, 1090–1108. 10.1037/pag0000389 31804114

[B23] Ghanbarpour T. Sahabeh E. Gustafsson A. (2022). Consumer response to online behavioral advertising in a social media context: the role of perceived ad complicity. Psychol. Market. 39, 1853–1870. 10.1002/mar.21703

[B24] Guinote A. (2007). Power affects basic cognition: Increased attentional inhibition and flexibility. J. Exp. Soc. Psychol. 43, 685–697. 10.1016/j.jesp.2006.06.008

[B25] Han W. J. (2025). The encoding strategy of rednote content marketing under 4I Theory-A Case Study of Beauty Notes. E-Commer. Lett. 14, 931–936. 10.12677/ecl.2025.1451364

[B26] Hargittai E. Gruber J. Djukaric T. Fuchs J. Brombach L. (2020). Black box measures? How to study people's algorithm skills. Inf. Commun. Soc. 23, 764–775. 10.1080/1369118X.2020.1713846

[B27] Hayes A. F. (2013). Introduction to Mediation, Moderation, and Conditional Process Analysis: A Regression-Based Approach. New York, NY: Guilford Press.

[B28] Hodges J. A. Trace C. B. (2023). Preserving algorithmic systems: a synthesis of overlapping approaches, materialities and contexts. J. Document. 79, 1380–1392. 10.1108/JD-09-2022-0204

[B29] Hong J. W. Chen R. W. (2022). Consciousness stimulation and rule imagination: tactical dependencies and practical paths for users resisting algorithm. J. Journal. Commun. Res. 29, 38–56.

[B30] Hu J. Wang R. (2024). Familiarity breeds trust? The relationship between dating app use and trust in dating algorithms via algorithm awareness and critical algorithm perceptions. Int. J. Hum. Comput. Interact. 40, 4596–4607. 10.1080/10447318.2023.2217014

[B31] Huangfu B. (2021). “playing the algorithm game”: discourse and mechanism of platform paternalism. Chin. J. Journal. Commun. 43, 111–129. 10.13495/j.cnki.cjjc.2021.11.006

[B32] Jiang Q. L. Xing F. B. (2025). Participatory algorithms: dual situations and resistance paradox of algorithmic actions. Journal. Mass Commun. 2, 58–71. 10.15897/j.cnki.cn51-1046/g2.20241216.001

[B33] Jin F. Tu P. (2018). The impact of helper group identity on the donation: moderated by power. Manage. J. 15, 1679–1685.

[B34] Julian J. W. Katz S. B. (1968). Internal versus external control and the value of reinforcement. J. Pers. Soc. Psychol. 8, 89–94. 10.1037/h0025318 5638027

[B35] Karizat N. Delmonaco D. Eslami M. Andalibi N. (2021). Algorithmic Folk theories and identity: how TikTok users co-produce knowledge of identity and engage in algorithmic resistance. Proc. ACM Hum. Comput. Interact. 5, 1–44. 10.1145/347604636644216

[B36] Lammers J. Galinsky A. D. Gordijn E. H. Otten S. (2008). Illegitimacy moderates the effects of power on approach. Psychol. Sci. 19, 558–564. 10.1111/j.1467-9280.2008.02123.x 18578845

[B37] Laukyte M. (2022). Averting enfeeblement and fostering empowerment: algorithmic rights and the right to good administration. Comput. Law Secur. Rev. 46:105718. 10.1016/j.clsr.2022.105718

[B38] Levenson H. (1981). “Differentiating among internality, powerful others, and chance,” in Research With the Locus of Control Construct (Academic Press), 15–63. 10.1016/B978-0-12-443201-7.50006-3

[B39] Li H. Chen S. Ni S. G. (2013). Escape decision-making based on intuition and deliberation under simple and complex judgment and decision conditions. Acta Psychol. Sin. 45, 94–103. 10.3724/SP.J.1041.2013.0009437113526

[B40] Liang Y. Liu H. (2013). Research on internet empowerment: process and issues. Southeast Commun. 4, 14–17. 10.13556/j.cnki.dncb.cn35-1274/j.2013.04.021

[B41] Lin H. (2025). Oscillation between resist and to not? Users' folk theories and resistance to algorithmic curation on Douyin. Social Media Soc. 11:14. 10.1177/20563051251313610

[B42] Lu V. University of Georgia. Music - MA. (2022). Music and China's Little Red Book. ProQuest Dissertations & Theses.

[B43] Lü W. Yang Y. Zhang Y. B. (2020). The impact of consumer perception of personalization on click intentions under AI personalized recommendation. Manage. Sci. 33, 44–57. 10.3969/j.issn.1672-0334.2020.05.004

[B44] Lv X. Chen Y. Guo W. (2022). Adolescents' algorithmic resistance to short video APP's recommendation: the dual mediating role of resistance willingness and resistance intention. Front. Psychol. 13:859597. 10.3389/fpsyg.2022.859597 35548508 PMC9083067

[B45] Magee J. C. Galinsky A. D. (2008). 8 Social hierarchy: the self-reinforcing nature of power and status. Acad. Manag. Ann. 2, 351–398. 10.5465/19416520802211628

[B46] Morimoto M. (2021). Privacy concerns about personalized advertising across multiple social media platforms in Japan: the relationship with information control and persuasion knowledge. Int. J. Advertis. 40, 431–451. 10.1080/02650487.2020.1796322

[B47] Park E. S. Hinsz V. B. Nickell G. S. (2015). Regulatory fit theory at work: prevention focus' primacy in safe food production. J. Appl. Soc. Psychol. 45, 363–373. 10.1111/jasp.12302

[B48] Peng L. (2021a). How to achieve “coexistence with algorithms” - algorithmic literacy and its two main aspects in the algorithm society. Explor. Confront. 3, 13–15+2.

[B49] Peng L. (2021b). The “prisoner” risk in the algorithm society. Global Media J. 8, 3–18. 10.16602/j.gmj.20210001

[B50] Rotter J. B. (1966). Generalized expectancies for internal versus external control of reinforcement. Psychol. Monogr. Gen. Appl. 80, 1–28. 10.1037/h0092976 5340840

[B51] Shin D. Park Y. J. (2019). Role of fairness, accountability, and transparency in algorithmic affordance. Comput. Human Behav. 98, 277–284. 10.1016/j.chb.2019.04.019

[B52] Shin D. Zhong B. Biocca F. A. (2020). Beyond user experience: what constitutes algorithmic experiences? Int. J. Inf. Manage. 52, 102061–11. 10.1016/j.ijinfomgt.2019.102061

[B53] Su H. (2023). Risks and avoidance in the public sphere of the algorithm society. Journal. Lover 9, 57–59. 10.16017/j.cnki.xwahz.2023.09.01427967229

[B54] Sun Y. Shan Y. Xie J. Chen K. Hu J. (2024). The relationship between social media information sharing characteristics and problem behaviors among chinese college students under recommendation algorithms the relationship between social media information sharing characteristics and problem behaviors among Chinese college students under recommendation algorithms. Psychol. Res. Behav. Manag. 17, 2783–2794. 10.2147/PRBM.S466398 39070065 PMC11283791

[B55] Tang Z. Duan J. W. Yan Y. Y. (2022). Dual domestication and human-technology hybridity: redefining algorithms from a domestication perspective. Acad. Res. 4, 56–60. 10.3969/j.issn.1000-7326.2022.04.010

[B56] Van T. Scully J. L. (2024). Unveiling algorithmic power: exploring the impact of automated systems on disabled people's engagement with social services. Disabil. Soc. 39, 3004–3029. 10.1080/09687599.2023.2233684

[B57] Velkova J. Kaun A. (2021). Algorithmic resistance: media practices and the politics of repair. Inf. Commun. Soc. 24, 523–540. 10.1080/1369118X.2019.1657162

[B58] Wallston K. A. Wallston B. S. Smith S. Dobbins C. J. (1987). Perceived control and health. Curr. Psychol. Res. Rev. 6, 5–25. 10.1007/BF02686633

[B59] Wan X. D. Xia Y. X. (2024). A study of users' willingness to shift information search field - based on the perspective of avoidance-based algorithmic resistance. New Media Res. 10, 1–8. 10.16604/j.cnki.issn2096-0360.2024.13.012

[B60] Wang X. (2023). Resisting in symbiosis: the technological mystique and subjective dilemma in the algorithm society. Southeast Acad. 4, 218–228. 10.13658/j.cnki.sar.2023.04.007

[B61] Wang X. Shang Q. (2024). How do social and parasocial relationships on TikTok impact the well-being of university students? The roles of algorithm awareness and compulsive use. Acta Psychol. 248:104369. 10.1016/j.actpsy.2024.104369 38936231

[B62] Weick M. Guinote A. (2008). When subjective experiences matter: power increases reliance on the ease of retrieval. J. Pers. Soc. Psychol. 94, 956–970. 10.1037/0022-3514.94.6.956 18505311

[B63] Willson M. (2017). Algorithms (and the) everyday. Inf. Commun. Soc. 20, 137–150. 10.1080/1369118X.2016.1200645

[B64] Yan Q. H. (2022).Emotional stratification in opinion expression online towards the issue of “the reform of individual income tax”: an analysis based on the measurement of emotional coverage. J. Soc. Sci. Hum. Normal Univ. 51, 144–156. 10.19503/j.cnki.1000-2529.2022.03.017

[B65] Yan Q. H. (2025). Research on the influence of situational algorithmic perception on Feedback behavior. Journal. Commun. Rev. 78, 100–117. 10.14086/j.cnki.xwycbpl.2025.01.008

[B66] Yi Q. L. (2022). Bridging medium theory and STS: materiality and new media studies. Nanjing J. Soc. Sci. 3, 96–107. 10.15937/j.cnki.issn1001-8263.2022.03.012

[B67] Yu G. M. Geng X. M. (2020). Technology empowerment and deconstruction of virtual idols in the age of artificial intelligence. J. Shanghai Jiaotong Univ. 28, 23–30. 10.13806/j.cnki.issn1008-7095.2020.01.006

[B68] Zakay D. Block R. A. (1997). Temporal cognition. Curr. Dir. Psychol. Sci. 6, 12–16. 10.1111/1467-8721.ep11512604

[B69] Zakay D. Block R. A. (2004). Prospective and retrospective duration judgments: an executive-control perspective. Acta Neurobiol. Exp. 64, 319–328. 10.55782/ane-2004-1516 15283475

[B70] Zarouali B. Boerman S. C. de Vreese C. H. (2021). Is this recommended by an algorithm? The development and validation of the algorithmic media content awareness scale (AMCA-scale). Telemat. Informatics 62:101607. 10.1016/j.tele.2021.101607

[B71] Zhang B.i, W. Zhang N. He L. (2024). Coping with homogeneous information flow in recommender systems: algorithmic resistance and avoidance. Int. J. Hum. Comput. Interact. 40, 6899–6912. 10.1080/10447318.2023.2267931

[B72] Zhao L. X. Lin C. (2023). Youth in the black box: algorithmic awareness, algorithmic attitude, and algorithmic manipulation among college students. China Youth Study 7, 20–30. 10.3969/j.issn.1002-9931.2022.07.003

[B73] Zhao S. D. Zhou Y. (2023). Imagined mediation: a study of college student users' attitudes towards Xiaohongshu's personalised recommendation algorithm. Media Sci. Technol. China 11, 91–95. 10.19483/j.cnki.11-4653/n.2023.11.018

